# From the ASPREE investigators: Response to Wittes et al.

**DOI:** 10.1177/17407745251344560

**Published:** 2025-07-06

**Authors:** John J McNeil, Andrew M Tonkin, Anne B Newman, Jeff D Williamson, Robyn L Woods, Andrew T Chan, Geoffrey A Donnan, Christopher M Reid, Mark R Nelson, Sara E Espinoza, Walter P Abhayaratna, Raj C Shah, Peter Gibbs, Michael E Ernst, Nigel P Stocks, Lawrence J Beilin, Brenda Kirpach, Joanne Ryan, Rory Wolfe, Anne M Murray, Karen L Margolis

As investigators of the ASPREE trial, we welcome informed critique of the study. However, the commentary by Wittes et al. referring to ‘an uninformative trial led to misinformed clinical guidelines’ has misinterpreted both the purpose of the study and its results.^1^ This response adds to the points already raised by Cleland and Anzar.^2^

To recap, ASPREE was a randomised double-blind, placebo-controlled clinical trial of low-dose aspirin in 19,114 older Australians and Americans.^3^ Its primary aim was to establish whether low-dose aspirin might increase the duration of disability-free survival in older adults free of manifest cardiovascular disease, dementia or physical disability. This endpoint was chosen because of its relevance to older individuals and to integrate both the posited favourable and unfavourable effects of aspirin.^4^ It was hypothesised that a reduction in cardiovascular disease events would provide a major part of the benefit, but it was also recognised that in an older population this might be offset by adverse effects of a long-term chronic medication.

After a median 4.7 years, the study was stopped after the DSMB and the sponsor determined that there was a negligible chance that a benefit from low-dose aspirin on disability-free survival (DFS) would become apparent if the trial continued to its scheduled end 6 months later. The result was unequivocal; there was no improvement in DFS among those randomised to low-dose aspirin.^5^ The non-significant trend for a reduction of vascular events was more than offset by an increase in all-cause mortality, coupled with an ongoing increase in serious bleeding.^6,7^ The cardiovascular outcomes were broadly similar to those of other contemporaneous trials.^8,9^

Following the publication of ASPREE and other major primary prevention trials, guideline committees in the US, Europe and elsewhere recommended against the routine use of aspirin in primary prevention of cardiovascular disease among older adults.^10,11^ These decisions were very much in accordance with the comment that ‘prescription should be based on an assessment of an individual’s benefit to risk’. Notwithstanding, Wittes et al. appear to have paid little attention to the adverse effects of low-dose aspirin which in ASPREE included significant increases in mortality, serious falls, anaemia and intracranial haemorrhage.^12–14^

Various other statements require correction. The combination of mortality and morbidities referred to by Wittes et al. was not a secondary prespecified endpoint and has never been reported on. Stroke outcomes adhered to the rigorous ‘TOAST’ principles.^15^ An analysis of major adverse cardiovascular events (MACE) was published as a non-prespecified outcome.^7^

While we agree that the prescription of aspirin should be based on the individual patient’s potential benefits and risks, this should be based on judgement that the absolute benefit substantially exceeds the absolute risks. Further analyses to determine if there exists a sub-group of the ASPREE cohort in whom benefit outweighs risk are ongoing, but as yet we have not been able to identify such a group.


John J McNeil and Andrew M Tonkin *School of Public Health and Preventive Medicine, Monash University, Melbourne, VIC, Australia*
Anne B Newman *Department of Epidemiology, University of Pittsburgh, Pittsburgh, PA, USA*
Jeff D Williamson *Section on Gerontology and Geriatric Medicine, Department of Internal Medicine, Wake Forest School of Medicine, Winston-Salem, NC, USA*
Robyn L Woods *School of Public Health and Preventive Medicine, Monash University, Melbourne, VIC, Australia*
Andrew T Chan *Clinical and Translational Epidemiology Unit, Division of Gastroenterology, Massachusetts General Hospital, Boston, MA, USA*
Geoffrey A Donnan *Melbourne Brain Centre, Department of Medicine and Neurology, The University of Melbourne, Melbourne, VIC, Australia*
Christopher M Reid *School of Public Health and Preventive Medicine, Monash University, Melbourne, VIC, Australia* *School of Population Health, Curtin University, Perth, Australia*
Mark R Nelson *Menzies Institute for Medical Research, University of Tasmania, Hobart, Tasmania, Australia*
Sara E Espinoza *Center for Translational Geroscience, Diabetes and Aging Center, Department of Medicine, Cedars-Sinai Medical Center, Los Angeles, CA, USA*
Walter P Abhayaratna *College of Health and Medicine, Australian National University, Canberra, Australian Capital Territory, Australia*
Raj C Shah *Department of Family and Preventive Medicine and the Rush Alzheimer’s Disease Center, Rush University, Chicago, IL, USA*
Peter Gibbs *Division of Personalised Oncology, Walter and Eliza Hall Institute of Medical Research, Melbourne, VIC, Australia*
Michael E Ernst *Department of Pharmacy Practice and Science, College of Pharmacy and Department of Family Medicine, Varver College of Medicine, The University of Iowa, Iowa City, IA, USA*
Nigel P Stocks *Discipline of General Practice, Adelaide Medical School, Adelaide University, Adelaide, SA, Australia*
Lawrence J Beilin *Medical School, Royal Perth Hospital Unit, The University of Western Australia, WA, Australia*
Brenda Kirpach *Berman Center for Outcomes and Clinical Research, Hennepin Healthcare Research Institute, Minneapolis, MN, USA*
Joanne Ryan and Rory Wolfe *School of Public Health and Preventive Medicine, Monash University, Melbourne, VIC, Australia*
Anne M Murray *Berman Center for Outcomes and Clinical Research, Hennepin Healthcare Research Institute, Minneapolis, MN, USA* *Department of Medicine, Hennepin Healthcare, Minneapolis, MN, USA*
Karen L Margolis *HealthPartners Research Foundation, Minneapolis, MN, USA*

